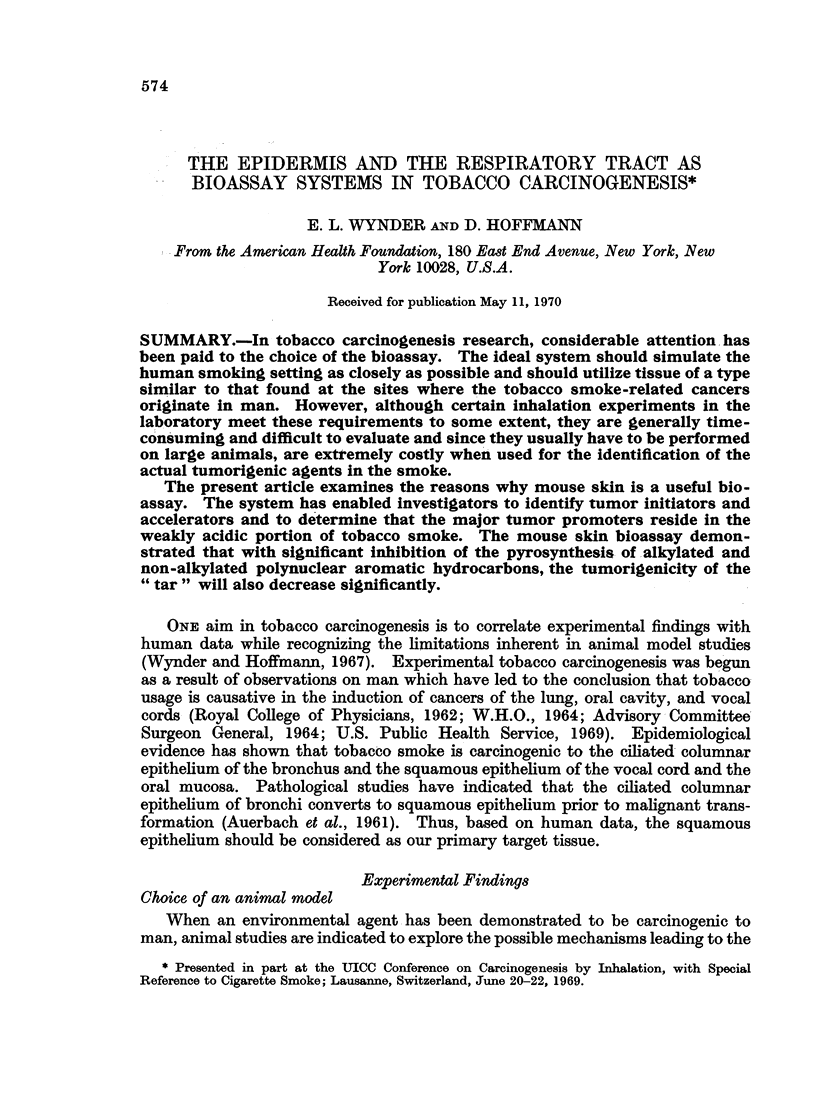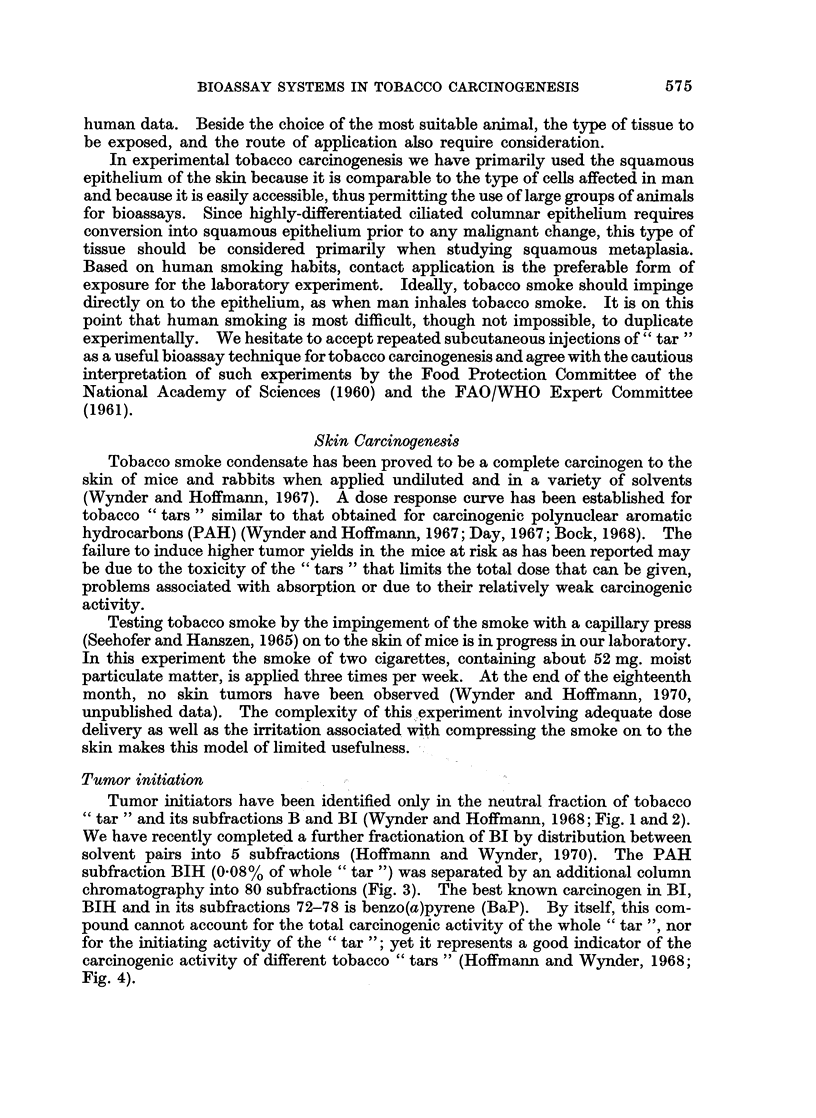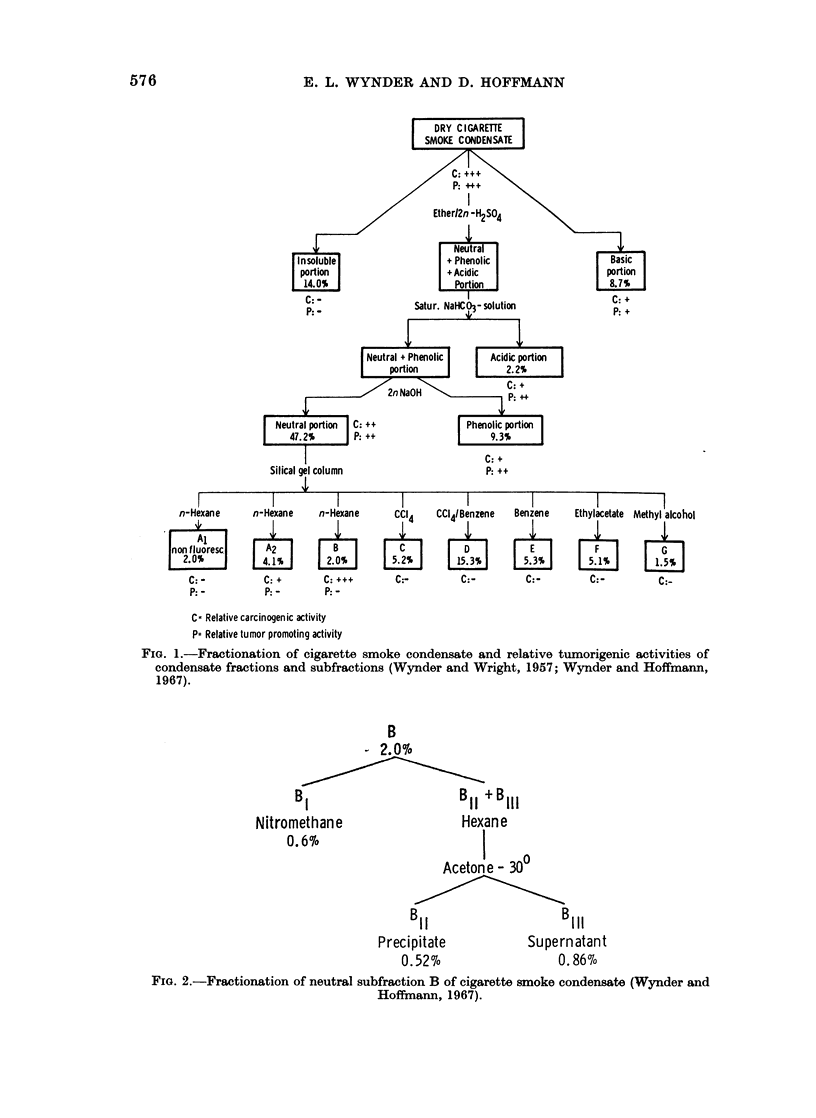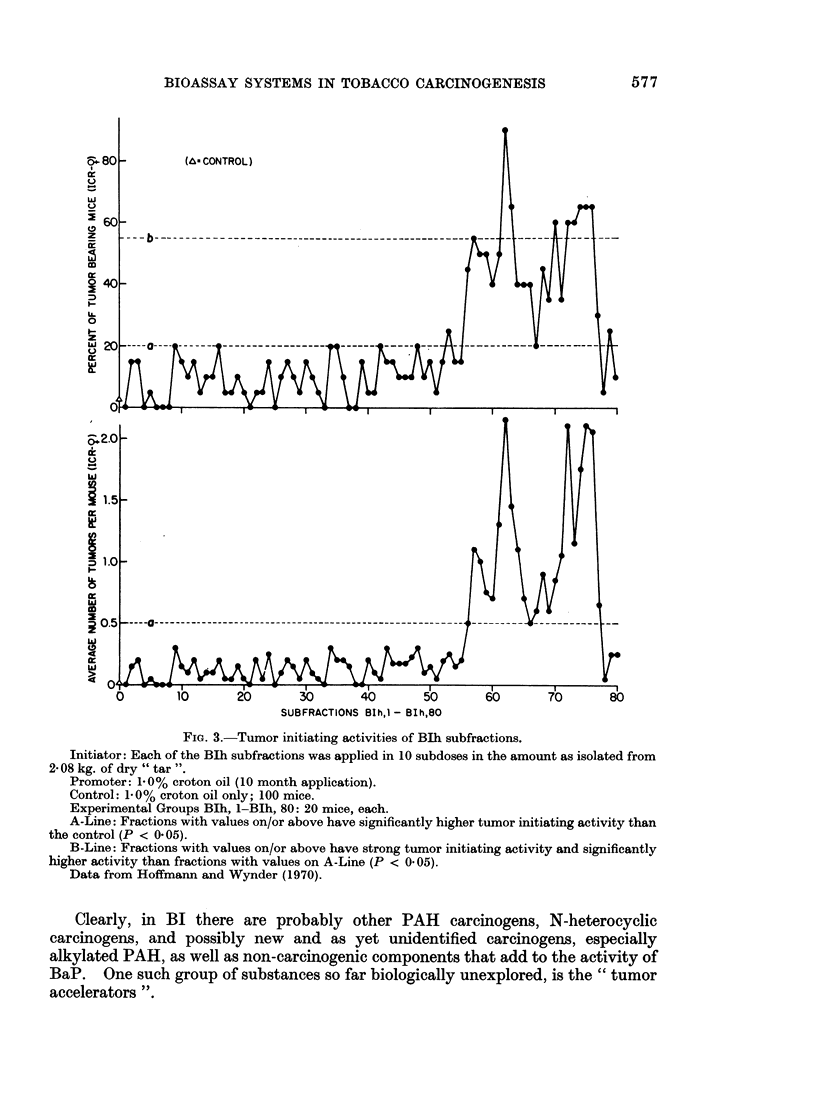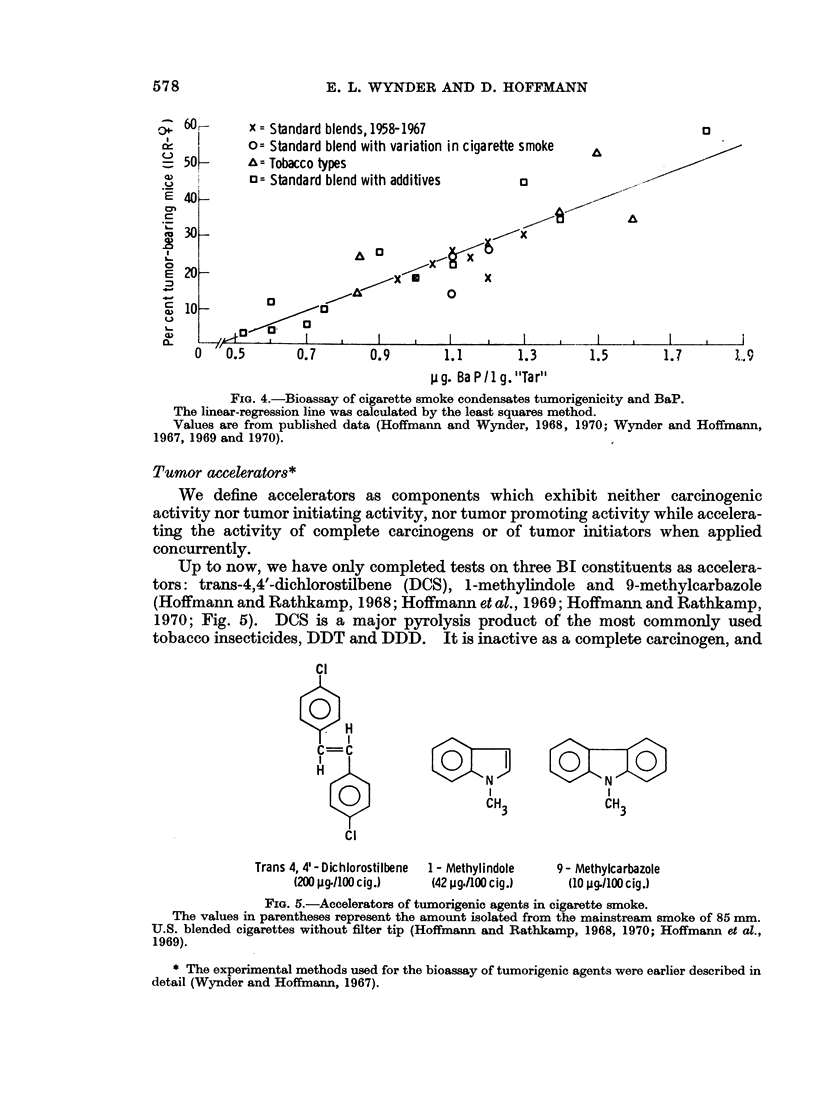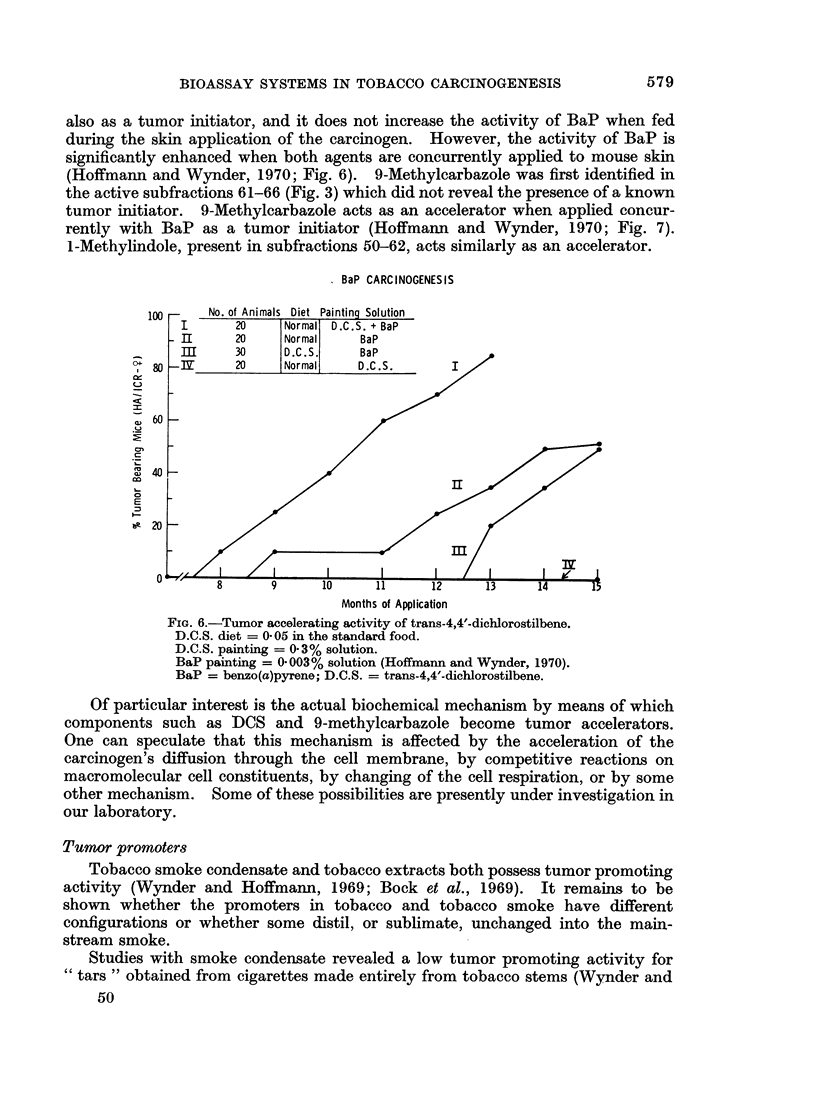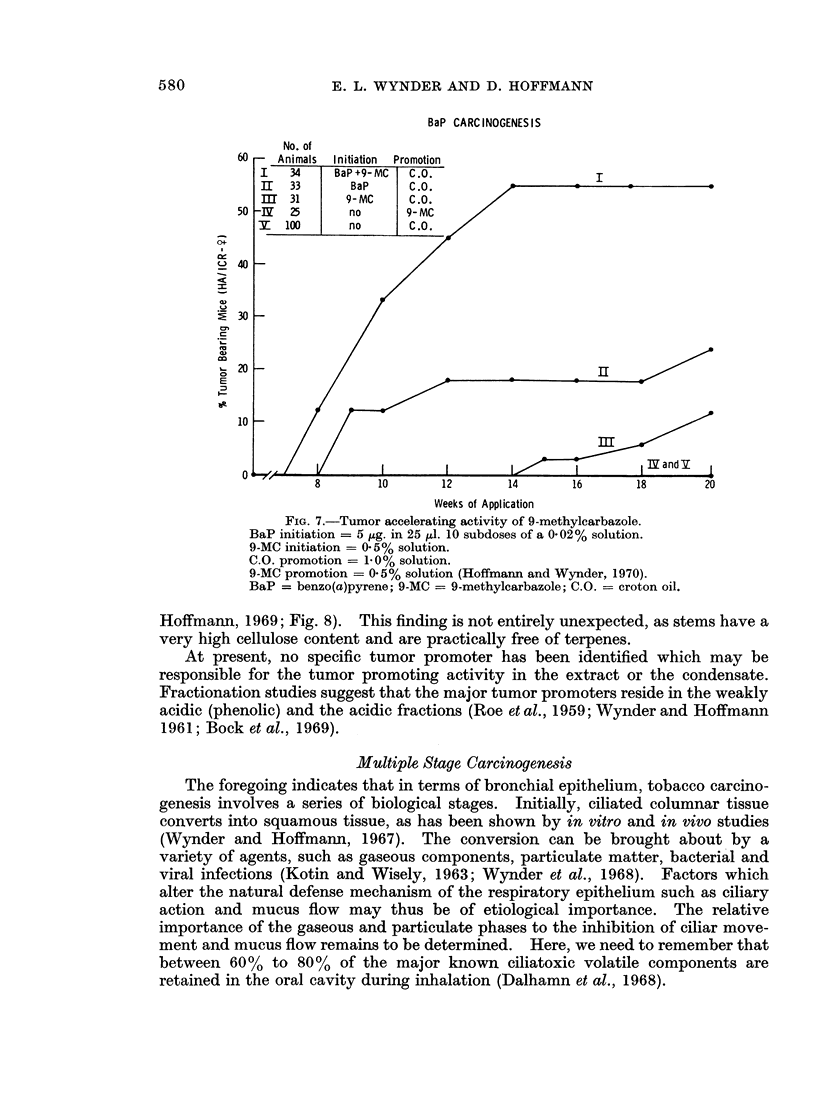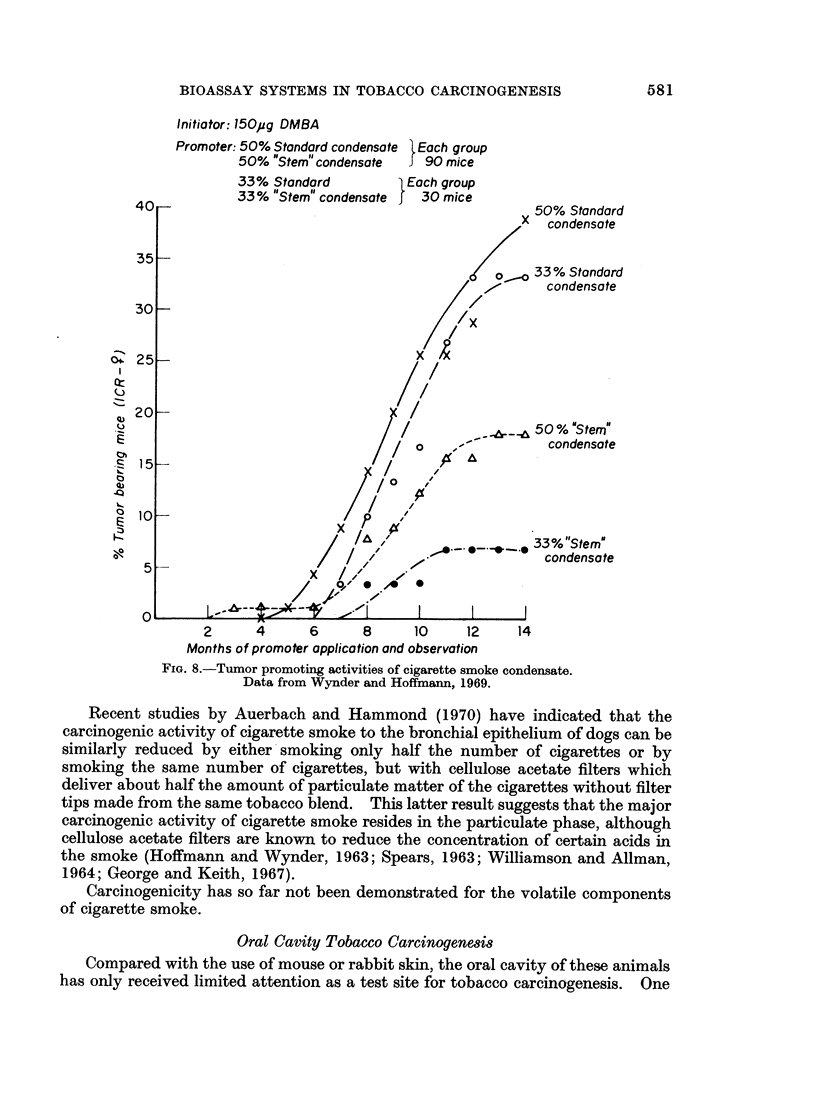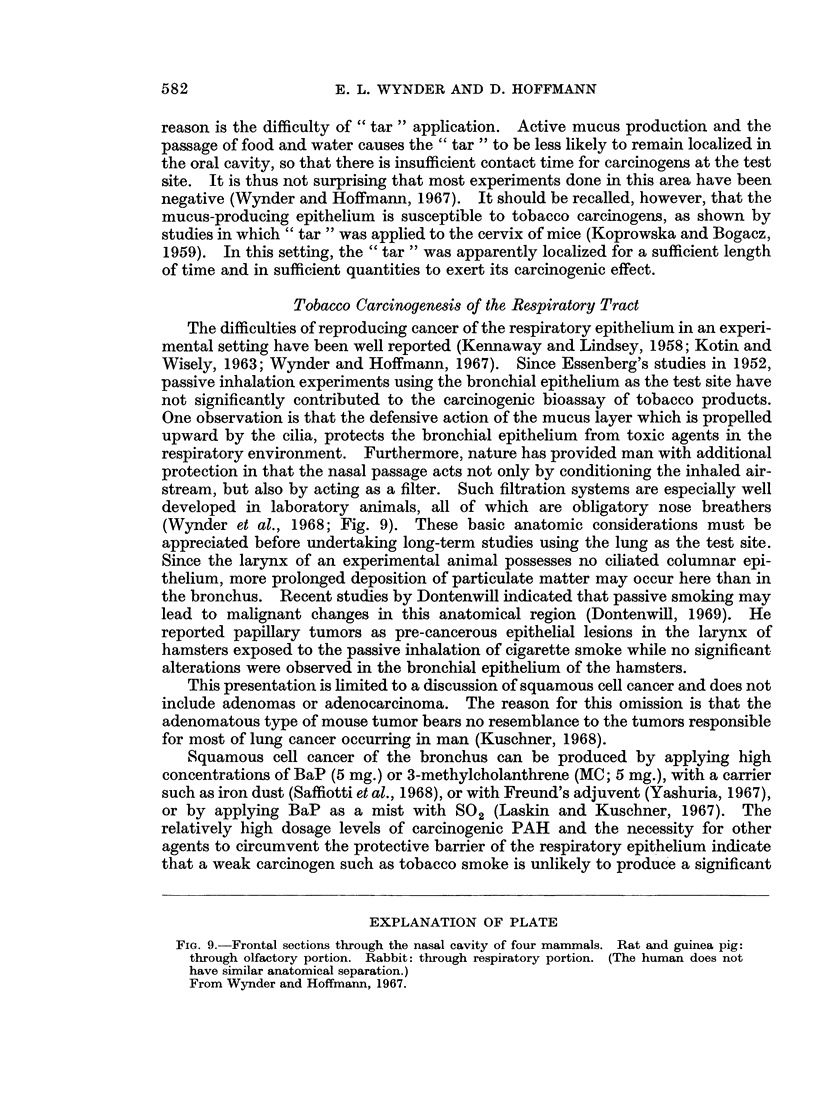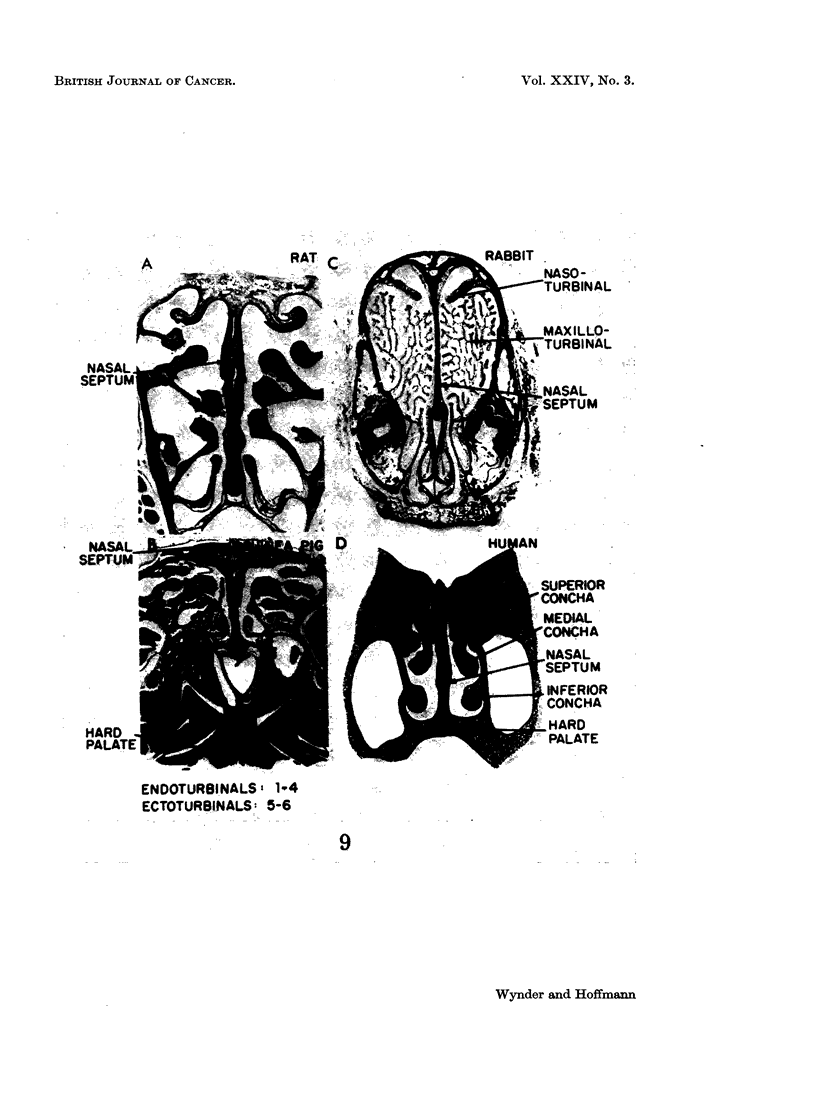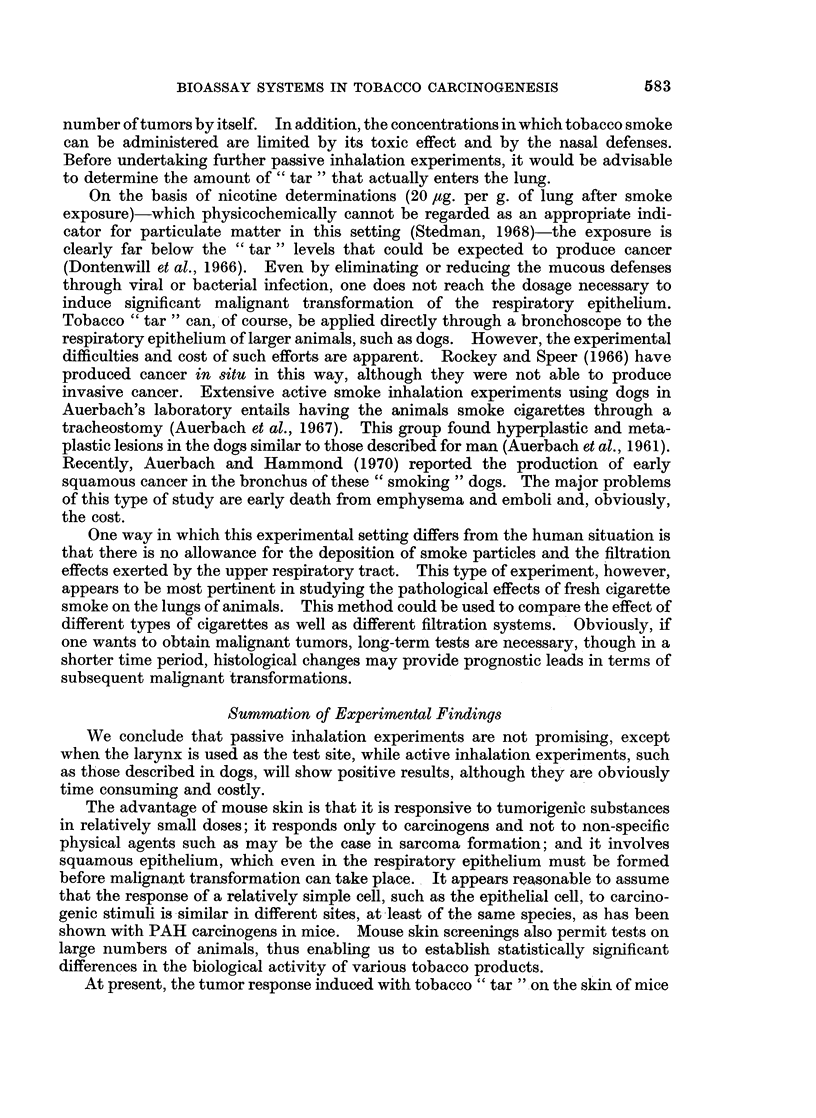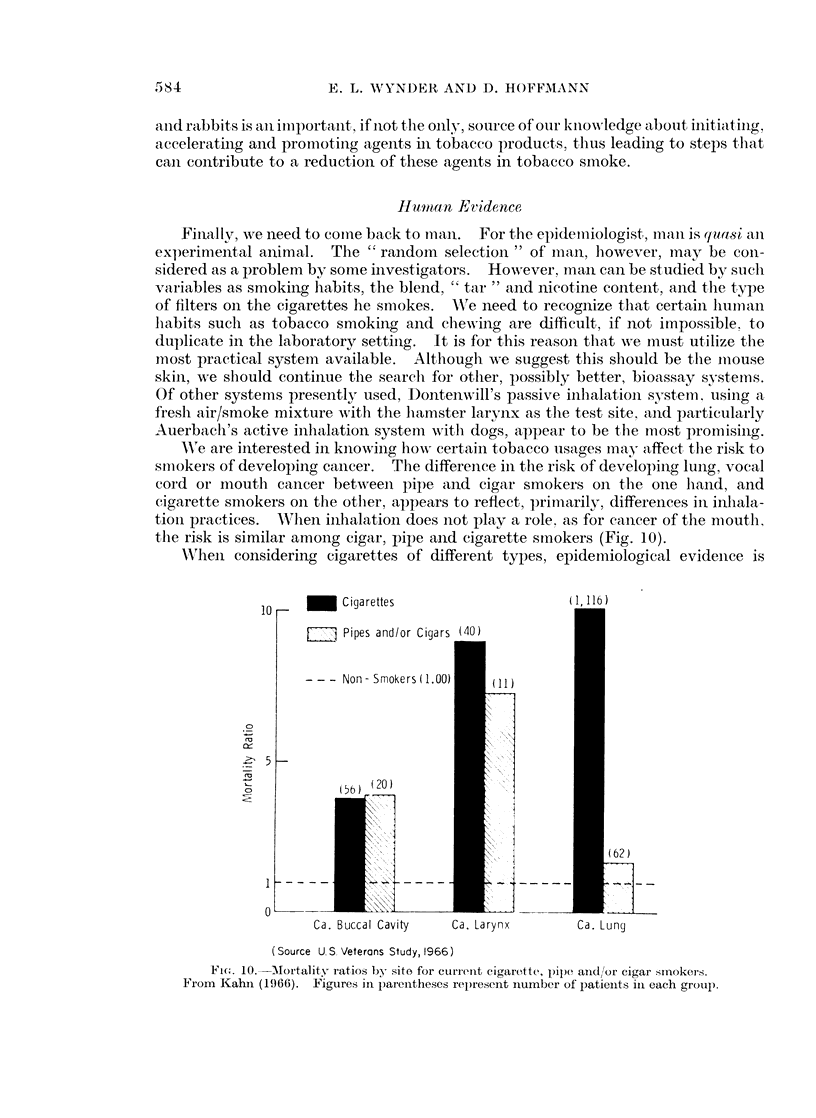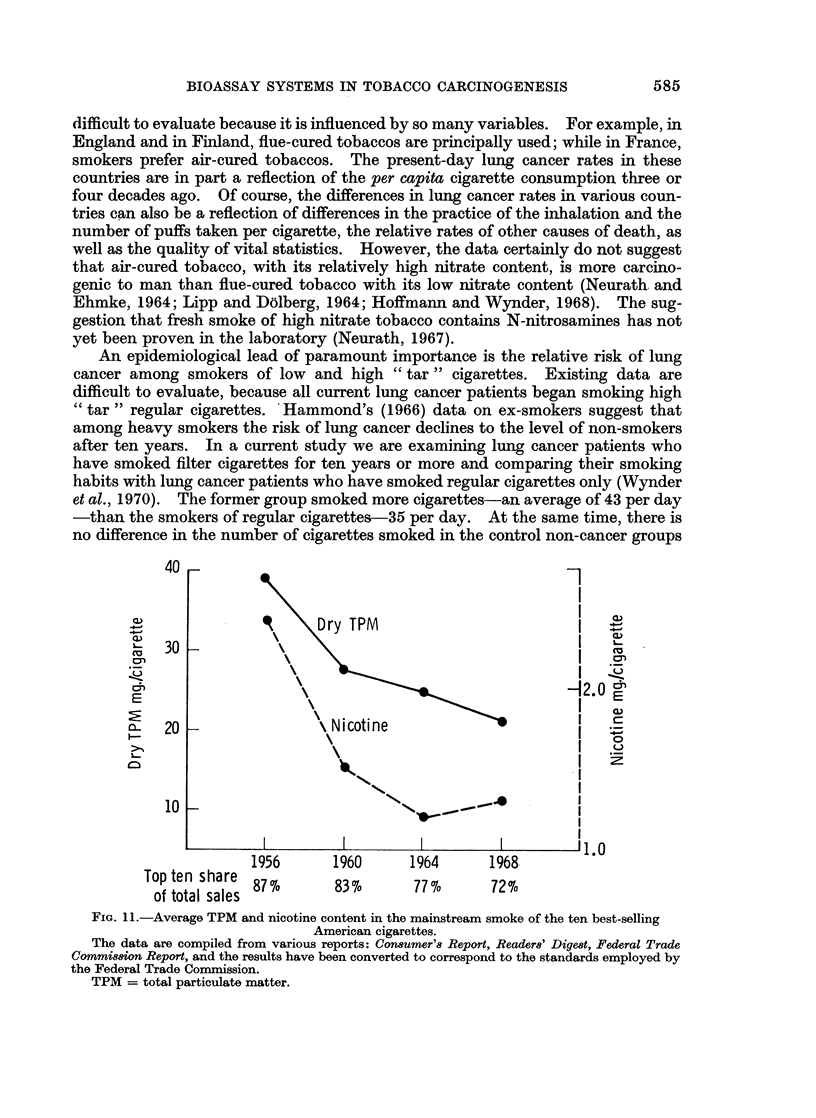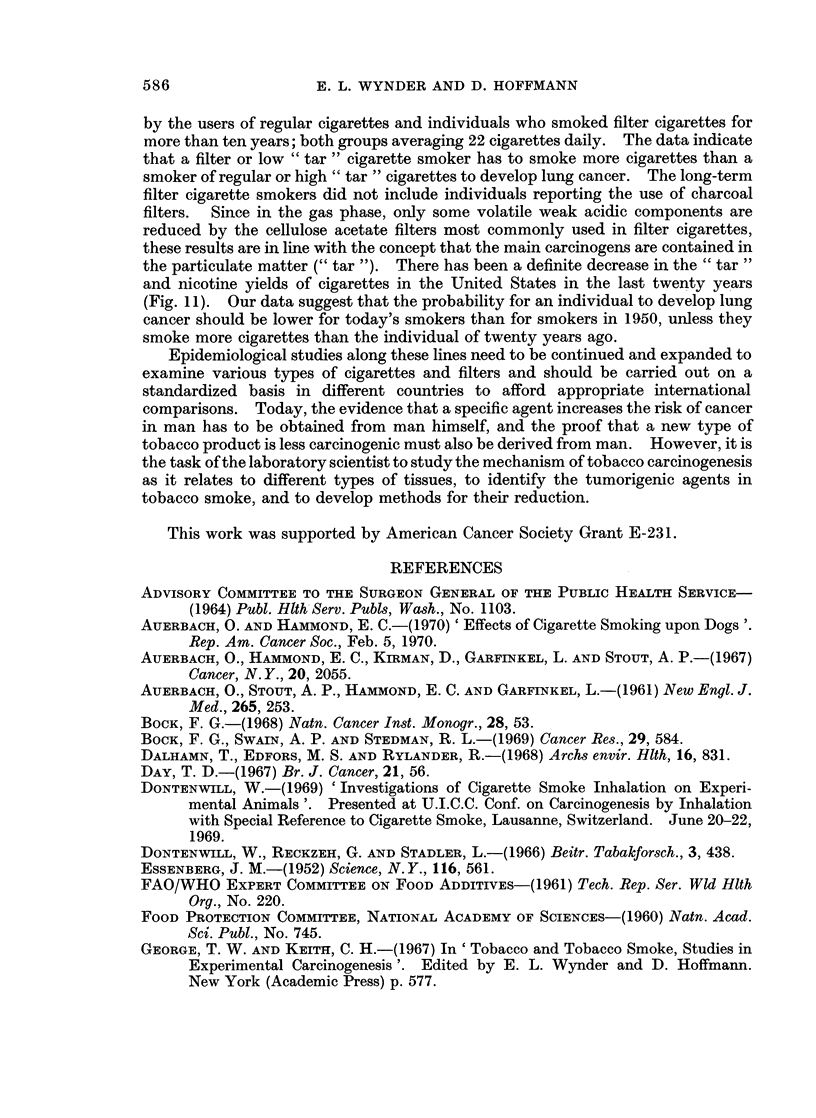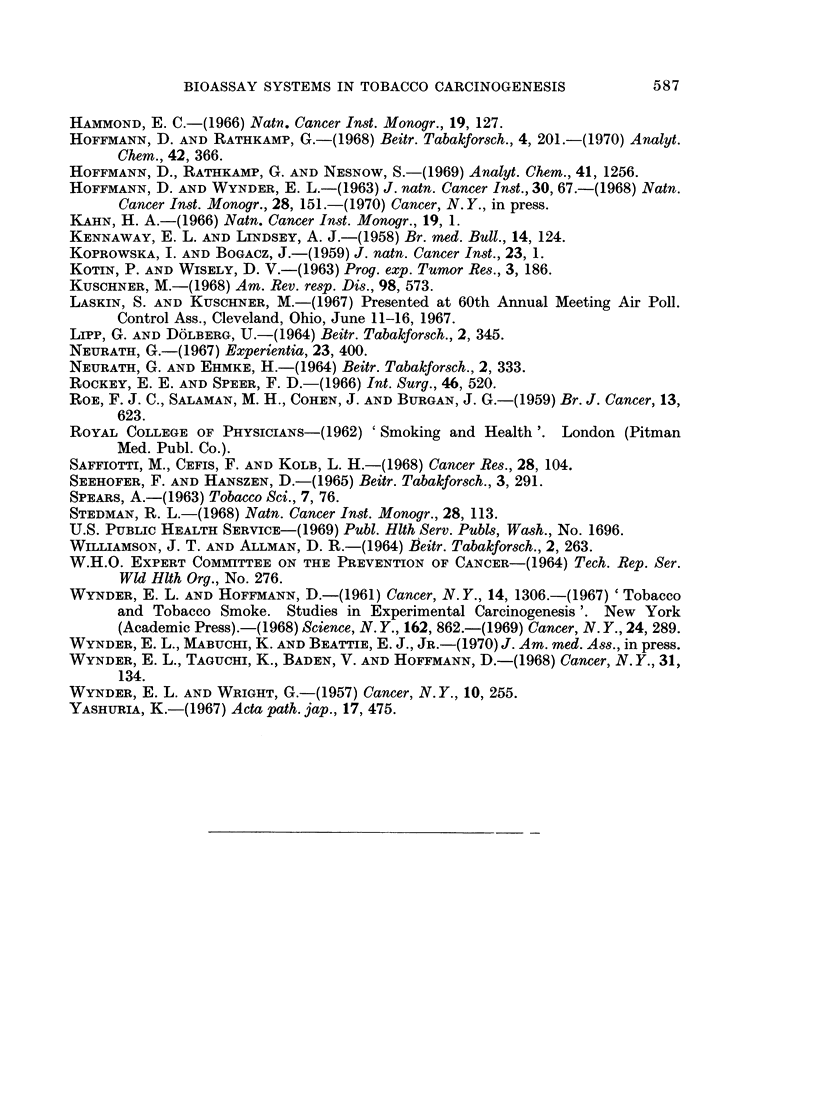# The Epidermis and the Respiratory Tract as Bioassay Systems in Tobacco Carcinogenesis*

**DOI:** 10.1038/bjc.1970.70

**Published:** 1970-09

**Authors:** E. L. Wynder, D. Hoffmann

## Abstract

**Images:**


### THE EPIDERMIS AND THE RESPIRATORY TRACT AS BIOASSAY SYSTEMS IN TOBACCO CARCINOGENESIS*

E. L. WYNDER AND D. HOFFMANN

*From the American Health Foundation,* 180 *East End Avenue, New York, New York* 10028, *U.S.A.*

Received for publication May 11, 1970

**SUMMARY.—In tobacco carcinogenesis research, considerable attention has been paid to the choice of the bioassay. The ideal system should simulate the human smoking setting as closely as possible and should utilize tissue of a type similar to that found at the sites where the tobacco smoke-related cancers originate in man. However, although certain inhalation experiments in the laboratory meet these requirements to some extent, they are generally time-consuming and difficult to evaluate and since they usually have to be performed on large animals, are extremely costly when used for the identification of the actual tumorigenic agents in the smoke.**

**The present article examines the reasons why mouse skin is a useful bioassay. The system has enabled investigators to identify tumor initiators and accelerators and to determine that the major tumor promoters reside in the weakly acidic portion of tobacco smoke. The mouse skin bioassay demonstrated that with significant inhibition of the pyrosynthesis of alkylated and non-alkylated polynuclear aromatic hydrocarbons, the tumorigenicity of the " tar " will also decrease significantly.**

ONE aim in tobacco carcinogenesis is to correlate experimental findings with human data while recognizing the limitations inherent in animal model studies (Wynder and Hoffmann, 1967). Experimental tobacco carcinogenesis was begun as a result of observations on man which have led to the conclusion that tobacco usage is causative in the induction of cancers of the lung, oral cavity, and vocal cords (Royal College of Physicians, 1962; W.H.O., 1964; Advisory Committee Surgeon General, 1964; U.S. Public Health Service, 1969). Epidemiological evidence has shown that tobacco smoke is carcinogenic to the ciliated columnar epithelium of the bronchus and the squamous epithelium of the vocal cord and the oral mucosa. Pathological studies have indicated that the ciliated columnar epithelium of bronchi converts to squamous epithelium prior to malignant transformation (Auerbach *et al.*, 1961). Thus, based on human data, the squamous epithelium should be considered as our primary target tissue.

### *Experimental Findings*

### *Choice of an animal model*

When an environmental agent has been demonstrated to be carcinogenic to man, animal studies are indicated to explore the possible mechanisms leading to the

* Presented in part at the UICC Conference on Carcinogenesis by Inhalation, with Special Reference to Cigarette Smoke; Lausanne, Switzerland, June 20–22, 1969.

human data. Beside the choice of the most suitable animal, the type of tissue to be exposed, and the route of application also require consideration.

In experimental tobacco carcinogenesis we have primarily used the squamous epithelium of the skin because it is comparable to the type of cells affected in man and because it is easily accessible, thus permitting the use of large groups of animals for bioassays. Since highly-differentiated ciliated columnar epithelium requires conversion into squamous epithelium prior to any malignant change, this type of tissue should be considered primarily when studying squamous metaplasia. Based on human smoking habits, contact application is the preferable form of exposure for the laboratory experiment. Ideally, tobacco smoke should impinge directly on to the epithelium, as when man inhales tobacco smoke. It is on this point that human smoking is most difficult, though not impossible, to duplicate experimentally. We hesitate to accept repeated subcutaneous injections of " tar " as a useful bioassay technique for tobacco carcinogenesis and agree with the cautious interpretation of such experiments by the Food Protection Committee of the National Academy of Sciences (1960) and the FAO/WHO Expert Committee (1961).

### *Skin Carcinogenesis*

Tobacco smoke condensate has been proved to be a complete carcinogen to the skin of mice and rabbits when applied undiluted and in a variety of solvents (Wynder and Hoffmann, 1967). A dose response curve has been established for tobacco " tars " similar to that obtained for carcinogenic polynuclear aromatic hydrocarbons (PAH) (Wynder and Hoffmann, 1967; Day, 1967; Bock, 1968). The failure to induce higher tumor yields in the mice at risk as has been reported may be due to the toxicity of the " tars " that limits the total dose that can be given, problems associated with absorption or due to their relatively weak carcinogenic activity.

Testing tobacco smoke by the impingement of the smoke with a capillary press (Seehofer and Hanszen, 1965) on to the skin of mice is in progress in our laboratory. In this experiment the smoke of two cigarettes, containing about 52 mg. moist particulate matter, is applied three times per week. At the end of the eighteenth month, no skin tumors have been observed (Wynder and Hoffmann, 1970, unpublished data). The complexity of this experiment involving adequate dose delivery as well as the irritation associated with compressing the smoke on to the skin makes this model of limited usefulness.

### *Tumor initiation*

Tumor initiators have been identified only in the neutral fraction of tobacco " tar " and its subfractions B and BI (Wynder and Hoffmann, 1968; Fig. 1 and 2). We have recently completed a further fractionation of BI by distribution between solvent pairs into 5 subfractions (Hoffmann and Wynder, 1970). The PAH subfraction BIH (0·08% of whole " tar ") was separated by an additional column chromatography into 80 subfractions (Fig. 3). The best known carcinogen in BI, BIH and in its subfractions 72–78 is benzo(*a*)pyrene (BaP). By itself, this compound cannot account for the total carcinogenic activity of the whole " tar ", nor for the initiating activity of the " tar "; yet it represents a good indicator of the carcinogenic activity of different tobacco " tars " (Hoffmann and Wynder, 1968; Fig. 4).

FIG. 1.—Fractionation of cigarette smoke condensate and relative tumorigenic activities of condensate fractions and subfractions (Wynder and Wright, 1957; Wynder and Hoffmann, 1967).

FIG. 2.—Fractionation of neutral subfraction B of cigarette smoke condensate (Wynder and Hoffmann, 1967).

FIG. 3.—Tumor initiating activities of BIh subfractions.

Initiator: Each of the BIh subfractions was applied in 10 subdoses in the amount as isolated from 2·08 kg. of dry " tar ".

Promoter: 1·0% croton oil (10 month application).

Control: 1·0% croton oil only; 100 mice.

Experimental Groups BIh, 1–BIh, 80: 20 mice, each.

A-Line: Fractions with values on/or above have significantly higher tumor initiating activity than the control ($P < 0{\cdot}05$).

B-Line: Fractions with values on/or above have strong tumor initiating activity and significantly higher activity than fractions with values on A-Line ($P < 0{\cdot}05$).

Data from Hoffmann and Wynder (1970).

Clearly, in BI there are probably other PAH carcinogens, N-heterocyclic carcinogens, and possibly new and as yet unidentified carcinogens, especially alkylated PAH, as well as non-carcinogenic components that add to the activity of BaP. One such group of substances so far biologically unexplored, is the " tumor accelerators ".

FIG. 4.—Bioassay of cigarette smoke condensates tumorigenicity and BaP.
The linear-regression line was calculated by the least squares method.
Values are from published data (Hoffmann and Wynder, 1968, 1970; Wynder and Hoffmann, 1967, 1969 and 1970).

*Tumor accelerators**

We define accelerators as components which exhibit neither carcinogenic activity nor tumor initiating activity, nor tumor promoting activity while accelerating the activity of complete carcinogens or of tumor initiators when applied concurrently.

Up to now, we have only completed tests on three BI constituents as accelerators: trans-4,4′-dichlorostilbene (DCS), 1-methylindole and 9-methylcarbazole (Hoffmann and Rathkamp, 1968; Hoffmann *et al.*, 1969; Hoffmann and Rathkamp, 1970; Fig. 5). DCS is a major pyrolysis product of the most commonly used tobacco insecticides, DDT and DDD. It is inactive as a complete carcinogen, and

FIG. 5.—Accelerators of tumorigenic agents in cigarette smoke.
The values in parentheses represent the amount isolated from the mainstream smoke of 85 mm. U.S. blended cigarettes without filter tip (Hoffmann and Rathkamp, 1968, 1970; Hoffmann *et al.*, 1969).

* The experimental methods used for the bioassay of tumorigenic agents were earlier described in detail (Wynder and Hoffmann, 1967).

also as a tumor initiator, and it does not increase the activity of BaP when fed during the skin application of the carcinogen. However, the activity of BaP is significantly enhanced when both agents are concurrently applied to mouse skin (Hoffmann and Wynder, 1970; Fig. 6). 9-Methylcarbazole was first identified in the active subfractions 61–66 (Fig. 3) which did not reveal the presence of a known tumor initiator. 9-Methylcarbazole acts as an accelerator when applied concurrently with BaP as a tumor initiator (Hoffmann and Wynder, 1970; Fig. 7). 1-Methylindole, present in subfractions 50–62, acts similarly as an accelerator.

FIG. 6.—Tumor accelerating activity of trans-4,4′-dichlorostilbene.
D.C.S. diet = 0·05 in the standard food.
D.C.S. painting = 0·3% solution.
BaP painting = 0·003% solution (Hoffmann and Wynder, 1970).
BaP = benzo(*a*)pyrene; D.C.S. = trans-4,4′-dichlorostilbene.

Of particular interest is the actual biochemical mechanism by means of which components such as DCS and 9-methylcarbazole become tumor accelerators. One can speculate that this mechanism is affected by the acceleration of the carcinogen's diffusion through the cell membrane, by competitive reactions on macromolecular cell constituents, by changing of the cell respiration, or by some other mechanism. Some of these possibilities are presently under investigation in our laboratory.

*Tumor promoters*

Tobacco smoke condensate and tobacco extracts both possess tumor promoting activity (Wynder and Hoffmann, 1969; Bock *et al.*, 1969). It remains to be shown whether the promoters in tobacco and tobacco smoke have different configurations or whether some distil, or sublimate, unchanged into the mainstream smoke.

Studies with smoke condensate revealed a low tumor promoting activity for " tars " obtained from cigarettes made entirely from tobacco stems (Wynder and

FIG. 7.—Tumor accelerating activity of 9-methylcarbazole.
BaP initiation = 5 μg. in 25 μl. 10 subdoses of a 0·02% solution.
9-MC initiation = 0·5% solution.
C.O. promotion = 1·0% solution.
9-MC promotion = 0·5% solution (Hoffmann and Wynder, 1970).
BaP = benzo(*a*)pyrene; 9-MC = 9-methylcarbazole; C.O. = croton oil.

Hoffmann, 1969; Fig. 8). This finding is not entirely unexpected, as stems have a very high cellulose content and are practically free of terpenes.

At present, no specific tumor promoter has been identified which may be responsible for the tumor promoting activity in the extract or the condensate. Fractionation studies suggest that the major tumor promoters reside in the weakly acidic (phenolic) and the acidic fractions (Roe *et al.*, 1959; Wynder and Hoffmann 1961; Bock *et al.*, 1969).

### *Multiple Stage Carcinogenesis*

The foregoing indicates that in terms of bronchial epithelium, tobacco carcinogenesis involves a series of biological stages. Initially, ciliated columnar tissue converts into squamous tissue, as has been shown by *in vitro* and *in vivo* studies (Wynder and Hoffmann, 1967). The conversion can be brought about by a variety of agents, such as gaseous components, particulate matter, bacterial and viral infections (Kotin and Wisely, 1963; Wynder *et al.*, 1968). Factors which alter the natural defense mechanism of the respiratory epithelium such as ciliary action and mucus flow may thus be of etiological importance. The relative importance of the gaseous and particulate phases to the inhibition of ciliar movement and mucus flow remains to be determined. Here, we need to remember that between 60% to 80% of the major known ciliatoxic volatile components are retained in the oral cavity during inhalation (Dalhamn *et al.*, 1968).

FIG. 8.—Tumor promoting activities of cigarette smoke condensate. Data from Wynder and Hoffmann, 1969.

Recent studies by Auerbach and Hammond (1970) have indicated that the carcinogenic activity of cigarette smoke to the bronchial epithelium of dogs can be similarly reduced by either smoking only half the number of cigarettes or by smoking the same number of cigarettes, but with cellulose acetate filters which deliver about half the amount of particulate matter of the cigarettes without filter tips made from the same tobacco blend. This latter result suggests that the major carcinogenic activity of cigarette smoke resides in the particulate phase, although cellulose acetate filters are known to reduce the concentration of certain acids in the smoke (Hoffmann and Wynder, 1963; Spears, 1963; Williamson and Allman, 1964; George and Keith, 1967).

Carcinogenicity has so far not been demonstrated for the volatile components of cigarette smoke.

### *Oral Cavity Tobacco Carcinogenesis*

Compared with the use of mouse or rabbit skin, the oral cavity of these animals has only received limited attention as a test site for tobacco carcinogenesis. One reason is the difficulty of " tar " application. Active mucus production and the passage of food and water causes the " tar " to be less likely to remain localized in the oral cavity, so that there is insufficient contact time for carcinogens at the test site. It is thus not surprising that most experiments done in this area have been negative (Wynder and Hoffmann, 1967). It should be recalled, however, that the mucus-producing epithelium is susceptible to tobacco carcinogens, as shown by studies in which " tar " was applied to the cervix of mice (Koprowska and Bogacz, 1959). In this setting, the " tar " was apparently localized for a sufficient length of time and in sufficient quantities to exert its carcinogenic effect.

### *Tobacco Carcinogenesis of the Respiratory Tract*

The difficulties of reproducing cancer of the respiratory epithelium in an experimental setting have been well reported (Kennaway and Lindsey, 1958; Kotin and Wisely, 1963; Wynder and Hoffmann, 1967). Since Essenberg's studies in 1952, passive inhalation experiments using the bronchial epithelium as the test site have not significantly contributed to the carcinogenic bioassay of tobacco products. One observation is that the defensive action of the mucus layer which is propelled upward by the cilia, protects the bronchial epithelium from toxic agents in the respiratory environment. Furthermore, nature has provided man with additional protection in that the nasal passage acts not only by conditioning the inhaled airstream, but also by acting as a filter. Such filtration systems are especially well developed in laboratory animals, all of which are obligatory nose breathers (Wynder *et al.*, 1968; Fig. 9). These basic anatomic considerations must be appreciated before undertaking long-term studies using the lung as the test site. Since the larynx of an experimental animal possesses no ciliated columnar epithelium, more prolonged deposition of particulate matter may occur here than in the bronchus. Recent studies by Dontenwill indicated that passive smoking may lead to malignant changes in this anatomical region (Dontenwill, 1969). He reported papillary tumors as pre-cancerous epithelial lesions in the larynx of hamsters exposed to the passive inhalation of cigarette smoke while no significant alterations were observed in the bronchial epithelium of the hamsters.

This presentation is limited to a discussion of squamous cell cancer and does not include adenomas or adenocarcinoma. The reason for this omission is that the adenomatous type of mouse tumor bears no resemblance to the tumors responsible for most of lung cancer occurring in man (Kuschner, 1968).

Squamous cell cancer of the bronchus can be produced by applying high concentrations of BaP (5 mg.) or 3-methylcholanthrene (MC; 5 mg.), with a carrier such as iron dust (Saffiotti *et al.*, 1968), or with Freund's adjuvent (Yashuria, 1967), or by applying BaP as a mist with $SO_2$ (Laskin and Kuschner, 1967). The relatively high dosage levels of carcinogenic PAH and the necessity for other agents to circumvent the protective barrier of the respiratory epithelium indicate that a weak carcinogen such as tobacco smoke is unlikely to produce a significant

---

EXPLANATION OF PLATE

FIG. 9.—Frontal sections through the nasal cavity of four mammals. Rat and guinea pig: through olfactory portion. Rabbit: through respiratory portion. (The human does not have similar anatomical separation.)
From Wynder and Hoffmann, 1967.

A
RAT
C
RABBIT
NASAL SEPTUM
NASO-TURBINAL
MAXILLO-TURBINAL
NASAL SEPTUM
1
5
2
3
6
4
NASAL SEPTUM
B
GUINEA PIG
D
HUMAN
1
5
6
2
3
4
SUPERIOR CONCHA
MEDIAL CONCHA
NASAL SEPTUM
INFERIOR CONCHA
HARD PALATE
HARD PALATE
ENDOTURBINALS: 1-4
ECTOTURBINALS: 5-6

9

number of tumors by itself. In addition, the concentrations in which tobacco smoke can be administered are limited by its toxic effect and by the nasal defenses. Before undertaking further passive inhalation experiments, it would be advisable to determine the amount of " tar " that actually enters the lung.

On the basis of nicotine determinations (20 $\mu$g. per g. of lung after smoke exposure)—which physicochemically cannot be regarded as an appropriate indicator for particulate matter in this setting (Stedman, 1968)—the exposure is clearly far below the " tar " levels that could be expected to produce cancer (Dontenwill *et al.*, 1966). Even by eliminating or reducing the mucous defenses through viral or bacterial infection, one does not reach the dosage necessary to induce significant malignant transformation of the respiratory epithelium. Tobacco " tar " can, of course, be applied directly through a bronchoscope to the respiratory epithelium of larger animals, such as dogs. However, the experimental difficulties and cost of such efforts are apparent. Rockey and Speer (1966) have produced cancer *in situ* in this way, although they were not able to produce invasive cancer. Extensive active smoke inhalation experiments using dogs in Auerbach's laboratory entails having the animals smoke cigarettes through a tracheostomy (Auerbach *et al.*, 1967). This group found hyperplastic and metaplastic lesions in the dogs similar to those described for man (Auerbach *et al.*, 1961). Recently, Auerbach and Hammond (1970) reported the production of early squamous cancer in the bronchus of these " smoking " dogs. The major problems of this type of study are early death from emphysema and emboli and, obviously, the cost.

One way in which this experimental setting differs from the human situation is that there is no allowance for the deposition of smoke particles and the filtration effects exerted by the upper respiratory tract. This type of experiment, however, appears to be most pertinent in studying the pathological effects of fresh cigarette smoke on the lungs of animals. This method could be used to compare the effect of different types of cigarettes as well as different filtration systems. Obviously, if one wants to obtain malignant tumors, long-term tests are necessary, though in a shorter time period, histological changes may provide prognostic leads in terms of subsequent malignant transformations.

### *Summation of Experimental Findings*

We conclude that passive inhalation experiments are not promising, except when the larynx is used as the test site, while active inhalation experiments, such as those described in dogs, will show positive results, although they are obviously time consuming and costly.

The advantage of mouse skin is that it is responsive to tumorigenic substances in relatively small doses; it responds only to carcinogens and not to non-specific physical agents such as may be the case in sarcoma formation; and it involves squamous epithelium, which even in the respiratory epithelium must be formed before malignant transformation can take place. It appears reasonable to assume that the response of a relatively simple cell, such as the epithelial cell, to carcinogenic stimuli is similar in different sites, at least of the same species, as has been shown with PAH carcinogens in mice. Mouse skin screenings also permit tests on large numbers of animals, thus enabling us to establish statistically significant differences in the biological activity of various tobacco products.

At present, the tumor response induced with tobacco " tar " on the skin of mice and rabbits is an important, if not the only, source of our knowledge about initiating, accelerating and promoting agents in tobacco products, thus leading to steps that can contribute to a reduction of these agents in tobacco smoke.

### *Human Evidence*

Finally, we need to come back to man. For the epidemiologist, man is *quasi* an experimental animal. The " random selection " of man, however, may be considered as a problem by some investigators. However, man can be studied by such variables as smoking habits, the blend, " tar " and nicotine content, and the type of filters on the cigarettes he smokes. We need to recognize that certain human habits such as tobacco smoking and chewing are difficult, if not impossible, to duplicate in the laboratory setting. It is for this reason that we must utilize the most practical system available. Although we suggest this should be the mouse skin, we should continue the search for other, possibly better, bioassay systems. Of other systems presently used, Dontenwill's passive inhalation system, using a fresh air/smoke mixture with the hamster larynx as the test site, and particularly Auerbach's active inhalation system with dogs, appear to be the most promising.

We are interested in knowing how certain tobacco usages may affect the risk to smokers of developing cancer. The difference in the risk of developing lung, vocal cord or mouth cancer between pipe and cigar smokers on the one hand, and cigarette smokers on the other, appears to reflect, primarily, differences in inhalation practices. When inhalation does not play a role, as for cancer of the mouth, the risk is similar among cigar, pipe and cigarette smokers (Fig. 10).

When considering cigarettes of different types, epidemiological evidence is

FIG. 10.—Mortality ratios by site for current cigarette, pipe and/or cigar smokers. From Kahn (1966). Figures in parentheses represent number of patients in each group.

difficult to evaluate because it is influenced by so many variables. For example, in England and in Finland, flue-cured tobaccos are principally used; while in France, smokers prefer air-cured tobaccos. The present-day lung cancer rates in these countries are in part a reflection of the *per capita* cigarette consumption three or four decades ago. Of course, the differences in lung cancer rates in various countries can also be a reflection of differences in the practice of the inhalation and the number of puffs taken per cigarette, the relative rates of other causes of death, as well as the quality of vital statistics. However, the data certainly do not suggest that air-cured tobacco, with its relatively high nitrate content, is more carcinogenic to man than flue-cured tobacco with its low nitrate content (Neurath and Ehmke, 1964; Lipp and Dölberg, 1964; Hoffmann and Wynder, 1968). The suggestion that fresh smoke of high nitrate tobacco contains N-nitrosamines has not yet been proven in the laboratory (Neurath, 1967).

An epidemiological lead of paramount importance is the relative risk of lung cancer among smokers of low and high " tar " cigarettes. Existing data are difficult to evaluate, because all current lung cancer patients began smoking high " tar " regular cigarettes. Hammond's (1966) data on ex-smokers suggest that among heavy smokers the risk of lung cancer declines to the level of non-smokers after ten years. In a current study we are examining lung cancer patients who have smoked filter cigarettes for ten years or more and comparing their smoking habits with lung cancer patients who have smoked regular cigarettes only (Wynder *et al.*, 1970). The former group smoked more cigarettes—an average of 43 per day —than the smokers of regular cigarettes—35 per day. At the same time, there is no difference in the number of cigarettes smoked in the control non-cancer groups

FIG. 11.—Average TPM and nicotine content in the mainstream smoke of the ten best-selling American cigarettes.

The data are compiled from various reports: *Consumer's Report*, *Readers' Digest*, *Federal Trade Commission Report*, and the results have been converted to correspond to the standards employed by the Federal Trade Commission.

TPM = total particulate matter.

by the users of regular cigarettes and individuals who smoked filter cigarettes for more than ten years; both groups averaging 22 cigarettes daily. The data indicate that a filter or low " tar " cigarette smoker has to smoke more cigarettes than a smoker of regular or high " tar " cigarettes to develop lung cancer. The long-term filter cigarette smokers did not include individuals reporting the use of charcoal filters. Since in the gas phase, only some volatile weak acidic components are reduced by the cellulose acetate filters most commonly used in filter cigarettes, these results are in line with the concept that the main carcinogens are contained in the particulate matter (" tar "). There has been a definite decrease in the " tar " and nicotine yields of cigarettes in the United States in the last twenty years (Fig. 11). Our data suggest that the probability for an individual to develop lung cancer should be lower for today's smokers than for smokers in 1950, unless they smoke more cigarettes than the individual of twenty years ago.

Epidemiological studies along these lines need to be continued and expanded to examine various types of cigarettes and filters and should be carried out on a standardized basis in different countries to afford appropriate international comparisons. Today, the evidence that a specific agent increases the risk of cancer in man has to be obtained from man himself, and the proof that a new type of tobacco product is less carcinogenic must also be derived from man. However, it is the task of the laboratory scientist to study the mechanism of tobacco carcinogenesis as it relates to different types of tissues, to identify the tumorigenic agents in tobacco smoke, and to develop methods for their reduction.

This work was supported by American Cancer Society Grant E-231.

### REFERENCES

Advisory Committee to the Surgeon General of the Public Health Service—(1964) *Publ. Hlth Serv. Publs, Wash.*, No. 1103.

Auerbach, O. and Hammond, E. C.—(1970) ' Effects of Cigarette Smoking upon Dogs '. *Rep. Am. Cancer Soc.*, Feb. 5, 1970.

Auerbach, O., Hammond, E. C., Kirman, D., Garfinkel, L. and Stout, A. P.—(1967) *Cancer, N.Y.*, **20**, 2055.

Auerbach, O., Stout, A. P., Hammond, E. C. and Garfinkel, L.—(1961) *New Engl. J. Med.*, **265**, 253.

Bock, F. G.—(1968) *Natn. Cancer Inst. Monogr.*, **28**, 53.

Bock, F. G., Swain, A. P. and Stedman, R. L.—(1969) *Cancer Res.*, **29**, 584.

Dalhamn, T., Edfors, M. S. and Rylander, R.—(1968) *Archs envir. Hlth*, **16**, 831.

Day, T. D.—(1967) *Br. J. Cancer*, **21**, 56.

Dontenwill, W.—(1969) ' Investigations of Cigarette Smoke Inhalation on Experimental Animals '. Presented at U.I.C.C. Conf. on Carcinogenesis by Inhalation with Special Reference to Cigarette Smoke, Lausanne, Switzerland. June 20–22, 1969.

Dontenwill, W., Reckzeh, G. and Stadler, L.—(1966) *Beitr. Tabakforsch.*, **3**, 438.

Essenberg, J. M.—(1952) *Science, N.Y.*, **116**, 561.

FAO/WHO Expert Committee on Food Additives—(1961) *Tech. Rep. Ser. Wld Hlth Org.*, No. 220.

Food Protection Committee, National Academy of Sciences—(1960) *Natn. Acad. Sci. Publ.*, No. 745.

George, T. W. and Keith, C. H.—(1967) In ' Tobacco and Tobacco Smoke, Studies in Experimental Carcinogenesis '. Edited by E. L. Wynder and D. Hoffmann. New York (Academic Press) p. 577.

HAMMOND, E. C.—(1966) *Natn. Cancer Inst. Monogr.*, **19**, 127.
HOFFMANN, D. AND RATHKAMP, G.—(1968) *Beitr. Tabakforsch.*, **4**, 201.—(1970) *Analyt. Chem.*, **42**, 366.
HOFFMANN, D., RATHKAMP, G. AND NESNOW, S.—(1969) *Analyt. Chem.*, **41**, 1256.
HOFFMANN, D. AND WYNDER, E. L.—(1963) *J. natn. Cancer Inst.*, **30**, 67.—(1968) *Natn. Cancer Inst. Monogr.*, **28**, 151.—(1970) *Cancer, N.Y.*, in press.
KAHN, H. A.—(1966) *Natn. Cancer Inst. Monogr.*, **19**, 1.
KENNAWAY, E. L. AND LINDSEY, A. J.—(1958) *Br. med. Bull.*, **14**, 124.
KOPROWSKA, I. AND BOGACZ, J.—(1959) *J. natn. Cancer Inst.*, **23**, 1.
KOTIN, P. AND WISELY, D. V.—(1963) *Prog. exp. Tumor Res.*, **3**, 186.
KUSCHNER, M.—(1968) *Am. Rev. resp. Dis.*, **98**, 573.
LASKIN, S. AND KUSCHNER, M.—(1967) Presented at 60th Annual Meeting Air Poll. Control Ass., Cleveland, Ohio, June 11–16, 1967.
LIPP, G. AND DÖLBERG, U.—(1964) *Beitr. Tabakforsch.*, **2**, 345.
NEURATH, G.—(1967) *Experientia*, **23**, 400.
NEURATH, G. AND EHMKE, H.—(1964) *Beitr. Tabakforsch.*, **2**, 333.
ROCKEY, E. E. AND SPEER, F. D.—(1966) *Int. Surg.*, **46**, 520.
ROE, F. J. C., SALAMAN, M. H., COHEN, J. AND BURGAN, J. G.—(1959) *Br. J. Cancer*, **13**, 623.
ROYAL COLLEGE OF PHYSICIANS—(1962) ‘Smoking and Health’. London (Pitman Med. Publ. Co.).
SAFFIOTTI, M., CEFIS, F. AND KOLB, L. H.—(1968) *Cancer Res.*, **28**, 104.
SEEHOFER, F. AND HANSZEN, D.—(1965) *Beitr. Tabakforsch.*, **3**, 291.
SPEARS, A.—(1963) *Tobacco Sci.*, **7**, 76.
STEDMAN, R. L.—(1968) *Natn. Cancer Inst. Monogr.*, **28**, 113.
U.S. PUBLIC HEALTH SERVICE—(1969) *Publ. Hlth Serv. Publs, Wash.*, No. 1696.
WILLIAMSON, J. T. AND ALLMAN, D. R.—(1964) *Beitr. Tabakforsch.*, **2**, 263.
W.H.O. EXPERT COMMITTEE ON THE PREVENTION OF CANCER—(1964) *Tech. Rep. Ser. Wld Hlth Org.*, No. 276.
WYNDER, E. L. AND HOFFMANN, D.—(1961) *Cancer, N.Y.*, **14**, 1306.—(1967) ‘Tobacco and Tobacco Smoke. Studies in Experimental Carcinogenesis’. New York (Academic Press).—(1968) *Science, N.Y.*, **162**, 862.—(1969) *Cancer, N.Y.*, **24**, 289.
WYNDER, E. L., MABUCHI, K. AND BEATTIE, E. J., JR.—(1970) *J. Am. med. Ass.*, in press.
WYNDER, E. L., TAGUCHI, K., BADEN, V. AND HOFFMANN, D.—(1968) *Cancer, N.Y.*, **31**, 134.
WYNDER, E. L. AND WRIGHT, G.—(1957) *Cancer, N.Y.*, **10**, 255.
YASHURIA, K.—(1967) *Acta path. jap.*, **17**, 475.